# Iodine Intake and Iodine Status in the Czech Republic – Past, Present, Future

**DOI:** 10.33549/physiolres.935493

**Published:** 2025-04-01

**Authors:** Radovan BÍLEK

**Affiliations:** 1 Institute of Endocrinology, Prague, Czech Republic

**Keywords:** Iodine intake, Iodine status, Hypothyroidism, Hyperthyroidism, Pregnancy

## Abstract

The physical and chemical properties of iodine, the importance of iodine for human health, iodine deficiency in the world and in Europe, the cycle of iodine in nature, values of iodine concentration in urine describing iodine deficiency, and the adequate or excessive supply of iodine to the body are presented in the work. The iodine intake of pregnant and lactating women and the state of iodine intake of these women in the Czech Republic are discussed. The history of iodine deficiency as well as the first mentions of the role of iodine in the thyroid gland and the first mentions of iodine prophylaxis in the world are explained. The present section describes the activities in the Czech Republic since the end of the Second World War, which contributed to the elimination of the iodine deficiency, including the establishment of the Interdepartmental Commission for Solving Iodine Deficiency. Population and other studies carried out in this period, which were related to the issue of iodine supply, are described. It was recorded that officially since 2004, the Czech Republic is among the countries where iodine deficiency is not currently a general problem. The future part is based on the Krakow appeal to leaders, politicians, scientists and officials, but also to the general population, to support actions leading to the elimination of iodine deficiency in Europe.

## Introduction

Iodine was discovered by the French chemist Bernard Courtois in 1811. It is a chemical element with the atomic number 53 and mass number from 108 to 144. Only iodine-127 with the atomic weight of 126.9 is stable, other iodine isotopes are radioactive. Iodine (I) is one electron short of a full octet and is hence a strong oxidizing agent. Elemental iodine forms diatomic molecules (I_2_), where two iodine atoms share a pair of electrons to achieve stable octet. Oxidation states of iodine are from −1 to +7. Iodide anion I^−^ is the strongest reducing agent among the stable halogens being the most easily oxidized back to diatomic I2. Iodine in the form of I_2_ is a solid with a melting point of 113.7 °C and it sublimes easily with gentle heat.

Iodine is essential in the biosynthesis of thyroid hormones that affect metabolic processes in the organism from the prenatal state to the elderly [1, 2, 3, 4]. Thyroid hormones influence the activity of practically all organs and are of irreplaceable importance in prenatal life during the normal brain development and the growth of the child [5,6,7]. Thyroid disease is accompanied by an increased incidence of metabolic, bone and cardiovascular diseases as well as increased mortality in these patients. Iodine deficiency is the most important risk factor for thyroid disease, and iodine deficiency is the most significant worldwide cause of unnecessary brain damage [8]. In recent decades, the incidence of pathophysiological disorders of the thyroid gland has increased, currently affecting approximately 2 billion people worldwide [9]. Thyroid problems and other endocrinopathies persist in all industrially developed countries including the Czech Republic and in countries where environmental pollution is observed [10].

An additional physiological role of iodine is related to the oldest terrestrial antioxidants used by living organisms due to its significant activity as a scavenger of reactive oxygen species [11]. Iodine oxidation to hypoiodite (IO-) by the effect of hydrogen peroxide under the catalysis of tissue-specific peroxidases (salivary-, gastric- and lacto-peroxidases) poses strong bacterial as well as antiviral and antifungal activity [12]. Iodine also has demonstrated an antineoplastic effect in various cell lines [13].

According to the World Health Organization (WHO) data, approximately 2 billion people currently live in conditions of iodine deficiency, a third being of school age, and these people have shown some type of thyroid ailment [10,14,15,16]. Of these, around 50 million develop clinical symptoms [17]. About 31 % of the world’s population has insufficient iodine intake, with the most severe deficiency occurring in Europe and Central and East Asia [18]. Historically iodine deficiency was seen in populations from inland regions (central Asia and Africa, central and eastern Europe, the central USA), mountainous areas (the Alps, Andes, Atlas, Himalayas) and those with frequent flooding (Southeast Asia) [3].

Europeans are increasingly affected by insufficient iodine intake [8]. In 2003, 56.9 % of the 435.5 million inhabitants in Europe had insufficient iodine intake (urinary iodine concentration <100 μg/L), including 59.9 % of the 42.2 million school children [15, 18]. In 2011, it was estimated that 44 % of the general population in Europe, i.e., 393 million inhabitants, had insufficient iodine intakes as evidenced by a urinary iodine concentration (UIC)<100 μg/L [19]. The number of iodine-deficient countries in the world has decreased to 32 in 2011, and 11 (34 %) are in Europe [20]. Large parts of Europe can be seen as mildly to moderately iodine deficient with only 27 % of European households having access to iodized salt [21]. The estimated annual economic benefit coming from the achievement of salt iodization in European countries is as much as €3.5 billion annually [22]. According to the United Nations Children’s Fund (UNICEF) data, some 88 % of the world’s population use iodinated salt. However, UNICEF also estimates that nearly 1 billion people still do not have access to iodized salt [23].

Iodine is contained in trace amounts in the geological bedrock, from where it migrates to the sea due to its volatile nature and the solubility of iodine compounds. The iodine originally contained in the earth’s surface was washed from the surface layers into the seas and oceans, and from there it reaches back to the land in the form of iodides oxidized by the effect of sunlight to molecular iodine escaping into the atmosphere. Atmospheric iodine is returned through rainfall or snowfall to the earth’s surface [24]. In the environment iodine is mainly present in the anionic forms (I^−^, IO3^−^, non-covalent I3^−^), while in the air iodine is found in the form of I_2_ associated with particles or volatile species, aerosol I^−^, and IO3^−^ [22].

The amount of iodine in soils depends on the geological substratum and the distance from the main reservoir of iodine, i.e. sea water, which contains 50–60 μg of iodine per litre [25,26]. In the marine environment, algae and phytoplankton are iodine hyperaccumulators helping to convert iodate (IO3^−^) into iodide (I^−^), the most absorbable form for terrestrial plants [27]. Soils have on average only about 3–12 mg/kg of total iodine [28,29]. Plants grown in iodine deficient soils have as low as 10 μg I/kg of dry weight, while plants grown in iodine rich soils have a concentration of 1 mg I/kg [25].

According to the World Health Organization (WHO), UNICEF and the lnternational Council for Control of lodine Deficiency Disorders (ICCIDD), urinary iodine concentration (UIC) below 20 μg/L denotes severe iodine deficiency, between 20–49 moderate, between 50–99 mild iodine deficiency, UlC between 100–199 is adequate iodine intake, UlC between 200–299 more than adequate, and UlC more than 300 μg/L is excessive iodine intake [1, 15, 18, 30]. UlC of pregnant women under 150 μg/L indicates an insufficient iodine intake, between 150–249 is adequate, between 250–499 is above the required level, and over 500 μg/L is excessive iodine intake [15, 18]. Median UIC equal to or exceeding 100 μg I/L is an adequate iodine intake for lactating women and children below 2 years of age [15,18]. The current (year 2024) WHO median UIC threshold defining adequate iodine intake (≥ 100 μg I/L) was originally defined for children and later extended to adults. However, the median UIC thresholds recommended for adults (100 μg I/L) and pregnant women (150 μg I/L) may underestimate the iodine intake and be inconsistent with the recommended average iodine intake of 150 μg I/day for adults and 250 μg I/day for pregnant women due to high urine volume in many European Region adult populations. The previous data are valid for school-age children, while epidemiological criteria are not currently defined for adults and pregnant women in the categories of severe or moderate iodine deficiency. Adequate iodine intake is => 100 μg/L for adults and excessive iodine intake for this population is also not defined [125].

The recommended daily intake of iodine for preschool children aged 0–12 months (upper limit not defined) and 1–5 years is 90 μg I/day (upper limit 200 μg I/day), for school children 6–12 years old it is 120 μg I/day (upper limit 300 μg I/day), for adolescents over 12 years old and adults it is equal to 150 μg I/day (upper limit 600 resp. 1100 μg I/day). For pregnant and lactating women, a higher iodine intake of 250 μg I/day is recommended [15, 18,125]. According to the 2014 the European Food Safety Authority (EFSA) panel, the upper limit of tolerated iodine intake for adults is 600 μg I/day [31]. The Institute of Medicine (USA) from 2001 gives a value of 1100 μg I/day [32].

The Czech Republic belongs to the iodine deficient area and the occurrence of endemic cretinism was recorded in the Czech Republic and Slovakia as early as at the beginning of the 20th century [33,34,35,36]. Cretinism is the most severe form of iodine deficiency characterized by mental retardation, deaf-mutism, stunted growth, delayed sexual maturation, and a variety of complications due to neurological abnormalities [6].

## Organification of iodine

A healthy adult body contains 15–20 mg of iodine [37], of which 70–80 % is in the thyroid gland, containing, on average, 5–15 mg of iodine in the normal human thyroid [4,38,39]. For the normal production of thyroid hormones, the thyroid gland absorbs 60–80 μg of iodine per day. Of this, a quarter is obtained by recycling endogenous iodine, and the rest comes from the diet. Unused iodine is rapidly excreted in the urine [40]. The presence of iodine in the organism is regulated by a multi-level feedback system, which handles possible fluctuations in iodine intake well [40]. Iodine must be taken from food and water, its main 2 chemical forms are iodide and iodate. More than 90 % of ingested iodine is absorbed in form of iodide into duodenum [3] and in the proximal small intestine [41]. Iodate is reduced in the proximal gut lumen to iodide [5, 40] which is taken up into the gut mucosa by a natrium iodide symporter (NIS). Some iodide crosses the gut mucosa by diffusion [41].

For healthy people, the optimal intake is 150 μg I/day. In pregnant and lactating women, due to the greater need for iodine, the daily intake of iodine corresponds to the value of 250 μg, which requires additional supplementation of 100 μg I/day using tablets of KI or by means of dietary supplements containing iodine [15]. Pregnant and lactating women and their children, as well as chronically ill people and people with non-traditional diets are at risk of iodine deficiency.

The results of the monitoring of dietary exposure of the State Health Institute - Center for Health, Nutrition and Food in 2018–2019 showed that the richest sources of iodine in the Czech Republic are in addition to iodized salt, also milk and dairy products, fish and meat products, pastries, bread, eggs and some types of mineral water (e.g., Vincentka, Hanácká kyselka, Zaječická bitter water, etc.). The average iodine intake during this period was 154 μg/person/day, which corresponds to a reference value of 150 μg I/day [42]. In 2021, 91 freely available food supplements containing iodine were identified on the Czech market. Some food supplements had a significantly higher iodine content, exceeding the limit of 150 μg I/day at recommended consumption (e.g. 1 drop of Kelp Vitabay contained 750 μg iodine) [43].

Absorbed iodide is transferred to the bloodstream and it is taken up by the thyroid gland through the action of natrium iodide symporter (NIS), glycoprotein present in basolateral plasma membrane of follicular epithelial cells or eliminated in urine [44, 45]. Inside the kidney, NIS expression was localized in the basolateral membrane of distal tubular cells and iodide is also reabsorbed by NIS observed in proximal and cortical collecting tubes [46]. Excretion or resorption of iodine will probably depend on the amount of iodine in the bloodstream [3].

The driving force of NIS in thyrocytes is the concentration gradient of Na+ ions generated by the Na+/K+ ATPase transporter [44]. NIS couples the inward movement of two Na^+^ ions with one I^−^ ion with active process driven by a Na^+^ gradient established by Na^+^/K^+^ adenosine triphosphatase [45]. Most of the iodide in the plasma is pumped out by NIS located in the thyrocyte. Accordingly, thyroid follicular cells may have an intracellular concentration 20- to 40-fold than that in plasma [45]. NIS is also found in the cells of the salivary glands, nasopharynx, in the mammary gland during lactation, in the placenta, in the lining of the stomach, intestines, in pancreas, thymus, choroid plexus, in the ciliary body of the eye and kidneys [45,47]. The expression and activity of NIS is influenced by the thyroid stimulating hormone (TSH), and additional thyroid restricted proteins such as pendrin on the apical side of the thyrocyte. The thyroid peroxidase facilitates the subsequent transport of I^−^ to the follicular lumen and its organification respectively [45]. If there is an insufficient intake of iodine, the levels of thyroid hormones will drop, mainly T4, less T3, and the production of TSH will increase. This leads to an increase in iodine uptake in the intestine by the effect of intestinal NIS. Thyroid NIS activity will also increase, leading to increased uptake of iodide from the plasma. An increase in renal NIS activity results in higher renal iodine retention. Conversely, with high iodine intake, these mechanisms are blocked [40].

Iodine is transferred to the lumen of thyroid follicles by pendrin situated on apical membrane and other transporters. The biosynthesis of thyroid hormones in the lumen is initiated by thyroid peroxidase (TPO), which uses H2O2 produced by ThOX1 and ThOX2 peroxidases to oxidize iodide to iodine radicals and incorporates it on specific tyrosine residues within thyroglobulin (TG) molecules secreted with thyrocytes [48]. After that, TPO couples two residues of diiodotyrosine (DIT) to form prohormone thyroxine (T4), and one monoiodotyrosine (MIT) to one DIT to form hormone triiodothyronine (T3), the main biologically active hormone of thyroid gland. Mature TG with thyroid hormones is reabsorbed from the lumen by micropinocytosis, and after proteolytic degradation by lysosomal enzymes the thyroid hormones are released to cytoplasm of thyrocytes. Uncoupled MIT and DIT residues are deiodinated by the iodotyrosine dehalogenase, a transmembrane protein localized mainly at the apical place of thyrocytes and involved in the intrathyroidal recycling of iodide. Thyroid hormones are transported outside of thyrocytes, mainly by monocarboxylate transporter 8, to the bloodstream [44]. The principal regulator of thyroid hormones biosynthesis is the pituitary TSH, which control the expression of the thyroid-specific genes involved in biosynthesis of thyroid hormones [48].

Selenium plays an important role in the production of glutathione, the body’s most powerful antioxidant. During the production of the thyroid hormones, hydrogen peroxide is produced in large quantities, and therefore high iodine in the absence of selenium can destroy the thyroid gland [49]. A family of selenium dependent enzymes (deiodinases D1 present in the liver, kidneys, thyroid, pituitary gland; D2 found mainly in the brain, brown fat, heart, pituitary gland; D3 occurring mainly in the brain, placenta, and liver) converts T4 to the main biologically active thyroid hormone T3 (specifically D2, non-specifically D1) and T4 to biologically inactive reverse T3 (specifically D3, non-specifically D1), and also convert T3 to T2 and further deiodinases the product of these reactions [50]. An adequate intake 70 μg Se/day was set for adults by the European Food Safety Authority (EFSA) in 2014 [51]. EFSA also established a tolerable upper intake 255 μg Se/day for adults in 2023 [52]. The most selenium in the Czech Republic is found in fish, egg, meat and cheese products. Median Se content in adults from the Czech Republic was 109 μg Se/L blood in 2009 and 84 μg Se/L blood in children in 2008 [53].

## Iodine deficiency

Too little a supply of iodine to the body leads to mental retardation, the formation of a goitre, hypothyroidism and a number of other diseases known as iodine deficiency disorders (IDD) [2]. Thyroid uptake of iodine varies substantially and increases when intake is low. Intestinal absorption efficiency of ingested iodine is more than 90 % [38]. In iodine-sufficient areas, the adult thyroid retains approximately 60 μg iodine per day to compensate for loss in faeces and sweat and also to maintain thyroid hormone synthesis [6]. A prevalence of goitres below 5 % was almost systematically observed for a UIC above 100 μg/L. This UIC corresponds to an approximate iodine intake of 150 μg/day in adults [38].

Mild iodine deficiency results in chronic thyroidal hyperstimulation. Correction of mild iodine deficiency decreases the prevalence of hyperthyroidism, which may be a life-threatening condition [125]. Chronic iodine deficiency may lead to compensatory thyroid hyperplasia with goitre due to an increase in concentration of TSH. The goitre is initially diffuse but later may become nodular with the appearance to autonomous nodules. Goitre increases the risk of thyroid cancer. Hypothyroidism (myxedema) also results from hormone deficiency [38].

IDD in prenatal life leads to miscarriages, stillbirths, congenital abnormalities including neurologic endemic cretinism (a neurological syndrome with severe mental retardation, spastic diplegic hearing defect and squinting) and congenital iodine deficiency syndrome previously known as endemic cretinism, which is characterized by mental deficiency, deafness, squinting, disorders of stance and gait and stunted growth due to hypothyroidism. There exists also an increased perinatal morbidity and mortality. IDD in neonates causes neonatal hypothyroidism, neonatal goitres, endemic mental retardation, low birth weight on average and a higher rate of congenital anomalies. Even mild and moderate iodine deficiency affects the intellectual development of the child. Neonatal TSH is elevated. IDD in children and adolescents are goitres, subclinical or clinical hypothyroidism and hyperthyroidism (multinodular goitres with autonomous nodules), impaired mental function, and impaired intellectual development in children. The reaction time to a stimulus increases, and the physical development of children deteriorates. IDD in adults are impairment of a neuro-intellectual development and the induction of the development of a goitre including multinodular goitres. There is also the development of iodine-induced hyperthyroidism in case of sudden iodine overload in previously severely iodine-deficient populations. In endemically deficient environments 10–15 IQ points is lost [15,18,54]. The single most preventable cause of intellectual disability is that of iodine deficiency [55].

Vegans are also a risk group, who are at risk of both iodine deficiency and complications from excessive iodine intake if they consume seaweed. By excluding all animal sources of iodine from the diet, the daily intake of iodine is reduced to 30 μg, moreover, the vegan diet contains a number of strumigens [56]. Cruciferous vegetables (i.e., broccoli, cabbage, kale, cauliflower) contain glucosinolates that have metabolites (thiocyanate and isothiocyanate) known to competitively inhibiting iodine transport and organification [57]. The metabolism of glucosinolates and cyanogenic glucosides leads to the production of cyanide and subsequently thiocyanate, and this group of goitrogens are found in certain vegetables such as cassava, sweet potatoes, maize, lima beans, bamboo shoots, linseed and sorghum. Additionally goitrogenic flavonoids in soy and millet may interfere with enzymatic activity of TPO involved in iodine metabolism [38]. Cooking prior to consumption can minimize goitrogenic effects [3]. Vegetarians should not develop an iodine deficiency if they consume enough dairy products and eggs [56].

The vast majority of territories in the Czech Republic are iodine deficient [58]. Among common foods, there is a high content of iodine in marine products of animal and plant origin. Consumption of these foods is insufficient in the Czech Republic and iodine must be artificially supplemented [59]. The main strategy for the control of iodine deficient disorders is salt iodization for its widespread consumption and the extremely low cost of iodization. The problem is with initiatives aimed at the reduction of overall salt consumption undertaken in Europe with the purpose of curbing cardiovascular disease rates in the region [15]. If the consumption of table salt in the Czech Republic drops from the current approximately 12 g/day to the recommended 5 g/day, the supply of iodine from this source will drop by more than half. The solution could be to increase the concentration of iodine in table salt and to consume more milk and milk products [58].

## Pregnant and lactating women

Iodine deficiency in pregnancy is a serious risk factor not only for the mother, but especially for the developing fetus and infants. The iodine needed for fetal synthesis of thyroid hormones leads to a total additional iodine intake rounded to 50 μg/day, i.e., an adequate intake of 200 μg/day is proposed for pregnant women. For lactating women the same adequate intake is proposed as for pregnant women, i.e., 200 μg/day [38]. Mild iodine deficiency in pregnancy may to lead to reduced IQ, as well as the development of cognitive and behavioral problems in childhood, due to the inadequate delivery of maternal thyroxine to the developing fetal brain [60]. The fetal thyroid gland reaches maturity close to the end of the first trimester and begins to secrete thyroid hormones by about week 16 [61]. The consequences of reduced iodine supply during the development of the brain and the entire organism of the fetus in the mother’s body and after delivery are permanent and cannot be completely removed. At the same time, even a mild iodine deficiency in the mother during pregnancy and lactation causes a decrease in intelligence and behavioral changes that persist throughout life [62,63]. According to WHO [15, 18], the single most preventable cause of intellectual disability is that of iodine deficiency [55]. Newborns and pregnant women remain very risky groups and their iodine saturation is borderline [64]. Premature newborns are particularly sensitive to iodine deficiency because they have a lower capacity to store it. Enteral nutrition usually falls short of the recommended iodine intake for preterm infants, which is 30–60 μg I/kg/day [65].

The percentage of newborns with TSH determined 48 to 72 hours after delivery between 5 and 15 mIU/L has already risen to 4.7 % in Bohemia and 2.9 % in Moravia in 2020 [64], which corresponds to a slight iodine deficit [40, 64]. A modest increase in neonatal TSH levels of 5–15 mIU/l in less than 3 % neonates is considered a normal iodine supply [64]. The monitoring of neonatal TSH from a dry drop of blood taken as part of screening for congenital hypothyroidism has been carried out in the Czech Republic on the initiative of prof. MUDr. O. Hníková, CSc. since 1996 [64].

Our older studies have shown that the results of iodine nutrition of pregnant women are alarming. In pairs, a full-term newborn and his mother had ioduria determined 5 days after birth in the years 1993 – 1995. The median ioduria of the newborns was 28 to 77 μg I/L, depending on the regions, and the median ioduria of the mothers corresponded to the median of their offspring. The examined pairs had moderate to severe iodine deficiency and it was recommended to give all pregnant and lactating mothers 100 μg I/day as a preventive measure and to enrich infant formula with iodine [66]. The urinary iodine concentrations were compared in 171 pregnant woman 2–8 weeks after an early spontaneous abortion with age-matched controls. The study was performed between April 2008 and December 2011. Women after spontaneous abortion residing in an iodine sufficient area suffer from mild iodine deficiency (median 92 μg I/L), which was significantly higher than in controls (median 117.8 μg I/L, n=181). Only 17 % of 171 women 2–8 weeks after an early spontaneous abortion have urinary iodine values greater than 150 μg/L [67]. In the period 2010–2015, only 30 % of a total of 750 examined pregnant women had iodide values higher than 150 μg/L [68] and thus met the WHO requirements for pregnant women [69, 70]. UIC was determined in 37 pregnant women and their newborns between December 2012 and March 2013. 20 out of 37 women took iodine supplements. Maternal UIC at the time of delivery was median 81.2 μg I/L in women without supplementation with comparison with women taking iodine supplements (median 101.9 μg I/L). Iodine supplementation during pregnancy affects mainly newborn UIC (median 102.9 μg I/L in women with iodine supplementation, n=20; median 87.8 μg I/L in women without iodine supplementation, n=17). Maternal UlC>150 μg/L at time of delivery was found in only 2 of 17 women without iodine supplementation [71]. Using ICP MS, it was found in 185 pregnant women examined between 2014 and 2015 that only 37 % of pregnant women are in the optimal range of ioduria between 150–300 μg I/L. In mild deficiency there were 21 % of women, and 6 % had a moderate iodine deficiency below 50 μg I/L [72].

The clinical impact of iodine deficiency diseases depends not only on the severity of the deficiency, but also on the clinical stage of development which the individual is in [5]. Adequate iodine intake during pregnancy prevents thyroid disease in both mothers and their fetuses and ensures appropriate physical and neurological development of the fetus and newborn [54,69,73]. Those forms of mental disorders that can be caused by a slight iodine deficiency are significant for a given state of iodine supply. Their result can be damage to the developing brain, affecting the intelligence of the fetus and its cognitive functions, in children they lead to a deterioration of the ability to learn, their intellectual development is limited, and in the longer term, the economic consequences are also manifested in the affected area [73]. In 2019, there were 0.599 million newborns suffering from IDD in Europe whose expected lifelong productivity losses would hit €4.4 billion [74]. Severe iodine deficiency in pregnant women is accompanied by a decrease in the IQ of their children by up to 13 points [15,18]. The necessary intake of iodine during pregnancy and lactation is higher in women (250 μg iodine per day) than in the general population (150 μg I/day) [15,18]. Although the risks of excessive iodine intake are relatively great, the consequences of excess iodine intake are often transient and less significant than the consequences of iodine deficiency [5].

Natural changes in thyroid function during pregnancy include increased placental production of human chorionic gonadotropin, which has a common alpha subunit with TSH and its potency is approximately 0.01 % of TSH [75]. There is also an increased production of oestrogens, which stimulate the biosynthesis of TBG in the liver. It is estimated that at 16–20 weeks of pregnancy, serum TBG doubles, and oestrogens also reduce its degradation in the circulation. An increased level of TBG results in a higher production of thyroid hormones in order to maintain the physiological concentration of the free fraction of thyroid hormones. An increase in the extrathyroidal distribution volume in pregnant women together with increased excretion of iodine by the kidneys due to a 50 % increase in glomerular filtration accompanied by a decrease in tubular resorption leads to an additional requirement for iodine intake in the body. The rise of D3 activity in the placenta in the 3rd trimester, which inactivates T4 and T3 to ineffective metabolites, again places increased demands on the biosynthesis of thyroid hormones by the mother [76]. Polyuria occurs in many cases in pregnant women [77]. The organism of pregnant women thus needs an approximately 50 % increase in iodine intake in order to maintain the physiological production of thyroid hormones both in the mother and in her foetus [54]. The current recommendation of the Czech Endocrinological Society is to use iodine in a dose of 150–200 μg I/day [64]. In women living in areas with sufficient iodine supply, thyroid volume increases by 10 % during pregnancy, women living in areas with iodine deficiency have thyroid volume increased by 20–40 % [78].

## Excess iodine

Together with TSH, iodine is one of the two main physiological regulators of thyroid function and volume. Unlike TSH, iodine acts negatively, i.e., an excess of iodine in the bloodstream reduces the response of the thyroid gland to TSH by inhibiting its own oxidation in the process of thyroid hormone biosynthesis (Wolf-Chaikoff effect) [79]. Iodine excess can disrupt thyroid function [80].

Excessive iodine intake (>200 μg I/L) is well tolerated to some extent, but in some susceptible individuals and those with preexisting thyroid disease, foetuses, neonates, and the elderly, or patients with other risk factors, excessive iodine intake may increase the risk of subclinical or overt thyroid disorders, which are related to both excessive (hyperthyroidism and autoimmune diseases) [81] and insufficient (hypothyroidism) [82] thyroid function following acute or chronic exposure [83, 84]. Excess intake of iodine is evidenced by the increased incidence of hyperfunction of the thyroid gland (thyrotoxicosis) and the activation of autoimmune processes in the thyroid gland, while the concentration of autoantibodies against thyroid peroxidase and thyroglobulin rises [58].

An acute Wolff-Chaikoff effect [79] means a transient inhibition of thyroid hormone synthesis lasting approximately 24 hours after intake of large amounts of iodide. It leads to the generation of intrathyroidal iodolactones, iodoaldehydes or iodolipids, which inhibit thyroid peroxidase activity [84,85]. Reduced intrathyroidal deiodinase activity may also contribute to decreased thyroid hormone synthesis [84,86]. The escape from the acute Wolf-Chaikoff effect over a period of 24 to 48 hours is associated with decreases in the natrium iodide symporter [84]. Failure to escape from the acute Wolff-Chaikoff effect may result in iodine-induced transient or permanent hypothyroidism in susceptible individuals with predisposing risk factors (patients with autoimmune thyroid disease, with Graves’ disease with a previous history of surgery, 131I treatment or antithyroid drug therapy, subacute or postpartum thyroiditis, type 2 amiodarone-induced thyrotoxicosis, etc.) [83,84]. Individuals living in regions of endemic iodine deficiency, in which goitrous disease is more common, may be at risk of iodine-induced hyperthyroidism (the Iodine-Basedow phenomenon) following salt iodization [83]. Euthyroid patients with nodular goitre in iodine-sufficient areas with excessive iodine supplementation are also at risk of the Iodine-Basedow phenomenon [84].

**Fig. 1:**
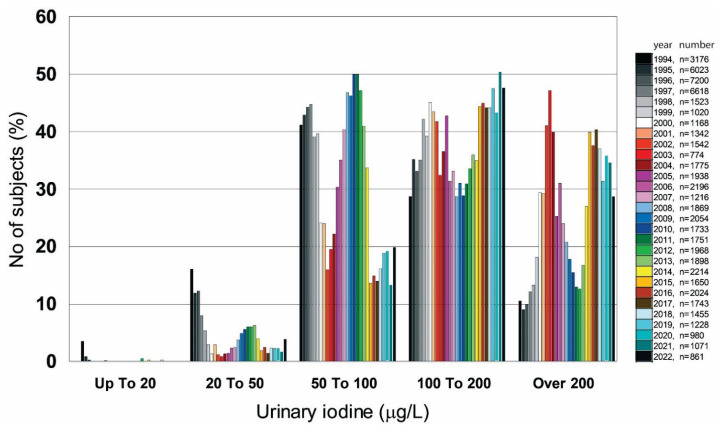
Combined histogram of iodine concentration in urine divided according to WHO [15] into groups with severe, moderate, mild deficiency, adequate, and more than adequate iodine intake from individuals visiting the Institute of Endocrinology in Prague, which also includes results from population studies conducted in the period 1994–2006. The years are marked in colour and the number of people is indicated.

Important exposure sources of iodine include milk and milk products. After 2000, an increase in iodine levels in milk caused by high doses of iodine in feed mixtures and mineral licks fed to cattle was recorded, which was also reflected in the increased ioduria of the population of the Czech Republic (Fig. 1) [87]. The iodine content of milk has continuously increased in average from 54 μg I/L in 1988 to 540 μg I/L in 2010 [88]. The problem of optimizing iodine in milk, when the iodine content varied between 50 and 700 μg I/L [58], must be addressed. The Interdepartmental Commission for Solving Iodine Deficiency (ICSID) in the Czech Republic made great efforts to reduce the iodine content in feed rations and the trend of increasing the content of iodine in milk was also noted within the European Union. The response to this fact was amended by the Regulation of the Commission of the European Community No. 1459/2005, which obligatorily reduced the limit of iodine in feed mixtures by half (max. 5 mg/kg of feed containing humidity 12 %) as the maximum allowed amount given to cows. The content of iodine in milk in the range of 100–200 μg/L is considered optimal from the point of view of supplying dairy cows with iodine.

Sources of iodine exposure in diet are mainly kelp (16–8165 μg I per gram), other sources are iodine-enriched vitamins and food supplements [84]. A source of excess iodine may be also medical products such as iodinated contrast agents, which contain approximately 13500 μg of free iodine (15 – 60 g of bound iodine) per CT scan, which can cause iodine-induced hypo- or hyper-thyroidism [84]. 54 adults were examined by CT scans after iodine administration of 34.6±6.0 g. The median of peak UIC 3519 (233 – 157500) μg I/L occurred at 1.1. weeks and normalized by 5.2 weeks following iodinated contrast media administration. Thyroid disfunction developed in 22 % of individuals [89]. Individuals treated with the drug amiodarone may receive 3–21 mg free iodine daily (75 mg I/200 mg tablet of amiodarone) [84]. Amiodarone-induced hypothyroidism appears to be more common in iodine sufficient areas, whereas amiodarone induced hyperthyroidism is seen more frequently in iodine-deficient regions [90]. Amiodarone-induced thyrotoxicosis has been categorized into type 1 and type 2. Type 1 is more prevalent among individuals with pre-existing thyroid disease living in regions of low iodine intake. Type 2 is a destructive thyroiditis in which thyrotoxicosis results from thyroid hormone release from the thyroid gland, and it usually occurs in patients with no history of thyroid disease [83]. The antiseptic agent consists of a stable chemical complex formed between very high concentrations of molecular iodine (I2) with the carrier molecule polyvinylpyrrolidone (povidone) bound in a non-covalent way [22]. This and other substances such as lithium, iodine-containing supplements, potassium iodide tablets and seaweed can also affect iodine transport. Chronic excessive iodine intakes may accelerate the development of subclinical thyroid disorders to overt hypothyroidism or hyperthyroidism, increase the incidence of autoimmune thyroiditis and increase the risk of thyroid cancer [35,91,92]. Disproportionately increased ioduria (>300 μg/L) has been noted especially in children, which is probably caused by increased consumption of milk and various supplemental mineral-vitamin mixtures containing iodine [58]. Although the risks of excessive iodine intake are relatively great, the consequences of excess iodine intake are often transient and less significant than the consequences of iodine deficiency [5].

## Indicators of iodine intake

A number of indicators, such as thyroid volume, urinary iodine content, thyrotropin, thyroglobulin, and thyroid hormones in blood have been discussed to determine the level of population iodine supply [15,18,30]. The most important indicator of iodine supply used in population studies is the urinary iodine concentration (UIC), which represents more than 90 % of the dietary intake and is therefore an excellent indicator of recent iodine intakes [38]. UIC levels, as determined by population representative studies, can be used as surrogate markers to monitor the effectiveness of IDD prevention programs [93]. The determination of iodine in urine affects the immediate state of iodine intake depending on food. With a sufficient number of samples, intra-individual variability and fluctuations in the iodine content of urine during the day are eliminated [15]. Thyroid volume and goitre prevalence are useful long-term clinical indicators of iodine status [38]. Thyroid volume reflects history of iodine intake but not current iodine status [4, 94]. The volume of the thyroid gland is not a good indicator of iodine supply, because when iodine deficiency is corrected for example with iodinated salt, it returns to the normal range only after a relatively long time [95]. TSH and thyroid hormones are not recommended as indicators of iodine intake in school children and adults because iodine deficiency leads to hypothyroidism, but the difference is not very large and there is often an overlap between individual values [30]. The TSH level is an important indicator of iodine supply in newly born infants [96]. Circulating thyroglobulin (TG) may be a sensitive indicator of iodine deficiency, and research is currently underway to further clarify the role of TG in iodine supply [1, 97, 98, 99, 100, 101]. The serum TG concentrations primarily reflect three factors: a) the mass of differentiated thyroid tissue present; b) any physical damage to or inflammation of the thyroid gland; and c) the magnitude of thyrotropin receptor stimulation [102]. The thyroid hyperplasia and goitre characteristic of iodine deficiency increases serum TG levels, and in this setting, the concentrations of serum TG reflects iodine nutrition over a period of weeks to months [4,15,18]. When determining TG, the standardization of kits from different manufacturers is particularly problematic. Our results show that the dependence of TG on I is U-shaped, where one half is at a concentration of 100–300 μg I/L. In insufficient iodine intake (UIC<100 μg I/L) and also in the subgroup with excessive iodine intake (UIC>300 μg I/L) the increase of TG is shown. Children and elderly people with any thyroid disorders are more sensitive to iodine deficiency than adults. In general TG serum concentrations higher than 40 μg/L should be an indicator for determining urinary iodine [98].

At our institute, iodine is determined using the alkaline ashing of urine, followed by a spectro-photometric determination of iodine in the form of iodide acting as a catalyst for the coupled oxidation-reduction Sandell-Kolthoff reaction between cerium and arsenic using brucine as a colorimetric indicator [36].

## Past

**Fig. 2:**
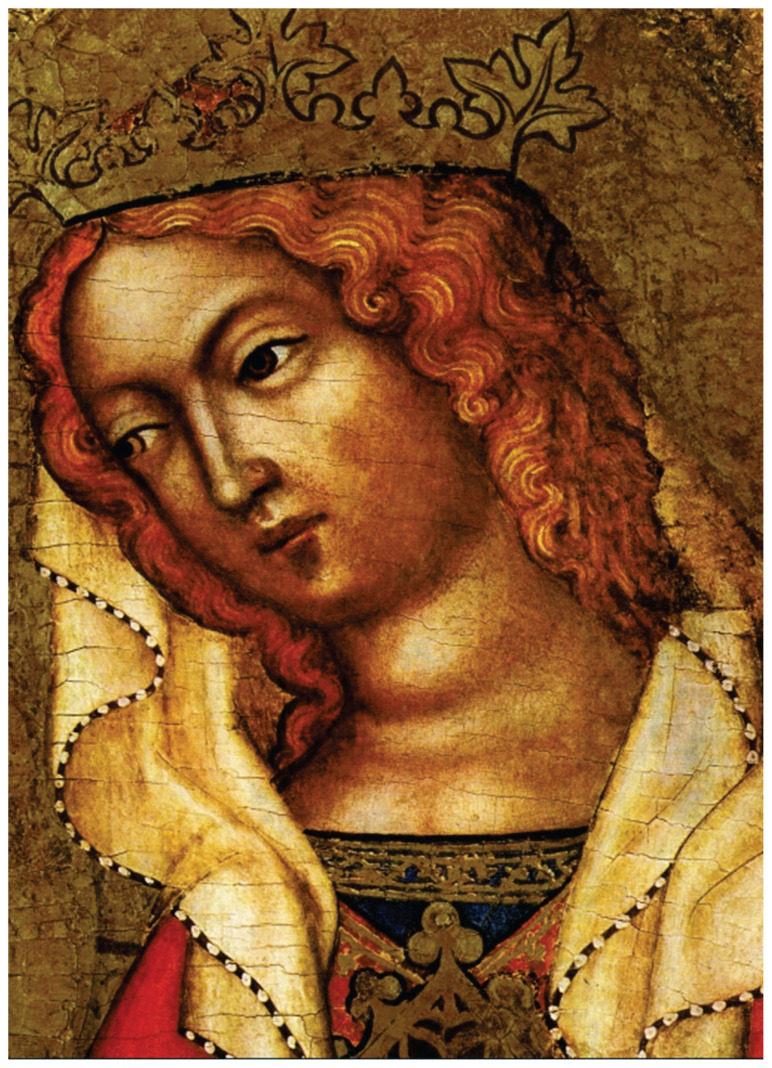
Detail of the Virgin Mary, with a visible goitre, in the painting *Adoration of the Three Kings* by the Master of the Vyšebrod Altar. The painter worked at the court of Emperor Charles IV in the middle of the 14th century.

**Fig. 3:**
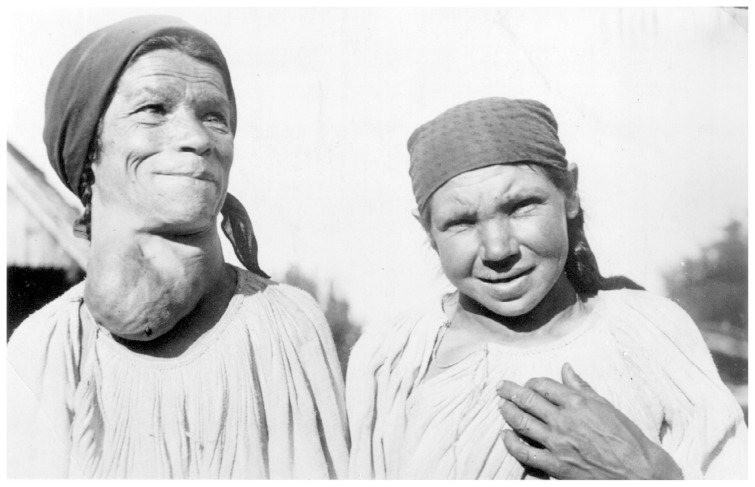
Doctor S. Vomely’s picture of 2 women from the central region of Transcarpathia with goitres and paracretinism in the beginning of the 20th century.

Iodine deficiency disorders belong to the history of Europe, especially in isolated and mountainous areas, but also in countries located in the central part of the continent where the population had a limited access to sea foods as a source of dietary iodine [103]. The goitre, a clinical manifestation of iodine deficiency, has apparently been known since ancient times and it was depicted in early Christian iconography [6]. It may be traced in medieval or even more recent paintings and sculptures, for example in statues of the Madonna (Fig. 2) in south Bohemia in the late 14th century [59]. A high prevalence of goitre was also recorded by military recruiting commissions of the former Austro-Hungarian army in the 19th century and in several medical papers from the first half of the 20th century [104]. Endemic cretinism even occurred in severely affected, mountainous areas (Fig. 3) until the first half of the last century [104].

Although iodine was discovered in 1811, it took another 100 years before its role in goitre reduction was recognized, and treatment of iodine deficiency was implemented [3]. In the late 1870s, research chemists and physicians in Europe identified the unifying characteristic of patients with goitres and cretinism as a thyroid that was low in iodine [6]. A landmark trial in the early 20th century definitively demonstrated that iodine supplementation could prevent what was then known as the "endemic goitre” [105]. American physicians Marine and Kimball probably first showed that iodine supplementation could reduce goitre in 1917 [106]. Prophylactic iodization of table salt started in Switzerland in 1922. In the first phase, 3.75 mg I/kg were added to the salt and this amount was doubled twice in 1962 and 1980. Until the time before prophylaxis, it was found in certain regions of Switzerland that 0.5 % of the inhabitants were cretins, almost 100 % of schoolchildren had large goitres and up to 30 % of young men were unfit for military service owing to a large goitre [107]. The introduction of iodized salt in Switzerland effectively eradicated iodine deficiency [107]. The occurrence of goitres with all of its side-effects were prevalent in the Czech Republic up to the first half of the twentieth century. The occurrence of goitres in women in certain regions reached as high as 80 % [35]. General practitioner from Wallachia Stanislav Vomela, MD, photographically documented the Carpathian region from Moravia to Subcarpathian Rus in the first half of the last century, where goitre and cretinism occurred (Fig. 3). The last cretin was born here in 1924 [62].

## Present

The current period is recorded from the end of World War II. The Czech Republic is among the countries with an early solution to iodine deficiency [34]. Thanks to doc. MUDr. K. Šilink, DrSc., founder and long-time director of the Institute of Endocrinology in Prague, a large-scale population study was organized in the period 1947–1953 focused on the examination of the thyroid gland and the mapping of iodine deficiency in individual parts of the Czech Republic [108]. Approximately 600,000 people participated in this study in Bohemia and Moravia. The results were not favourable [62]. It turned out that in most of the territory of the Czech Republic, the population is insufficiently supplied with iodine. The prevalence of goitres in women aged 21 to 50 years was on average about 60 % and in men of the same age about 30 %. Urinary iodine excretion corresponding to moderate iodine deficiency was recorded at 70 % from more than 5000 examined women [59]. Almost immediately, an iodine prophylaxis program was started, in the framework of which iodine was added to table salt in the form of potassium iodide in the amount of 5 ppm (5 mg I/kg of salt), in more affected districts in the amount of 12 ppm [59]. In 1966, Act No. 20/1966 Coll. and ČSN 580910 introduced mandatory iodization of table salt, during which iodine was added to the salt in the form of potassium iodide in the amount of 18 mg/kg. Table salt supplementation improved the situation regarding the incidence of diseases caused by an insufficient intake of iodine.

In the following period, less attention was paid to iodine prophylaxis and preventive programs aimed at healthy nutrition, which unfortunately resulted in an increase in the health risk of diseases caused by iodine deficiency. In the 1980s, there was an increase in the prevalence of goitres in all age categories of the population of the Czech Republic [34,58,109]. Prof. MUDr. V. Zamrazil, DrSc. from the Institute of Endocrinology organized extensive epidemiological studies focused on the evaluation of iodine deficiency and the state of the thyroid gland since the nineties of the last century [59, 110]. In the first half of the 90s of the last century, around 50 % of adults suffered from mild iodine deficiency; severe iodine deficiency occurred in around 12 % of men and 21 % of women, 7 % of boys and 10 % of girls [111]. The results of a random sample of the Prague population showed in 1992 that 13.2 % of boys, 15.2 % of men, 17 % of girls and 33.1 % of women suffered from moderate to severe iodine deficiency (<50 μg I/L). The volume of the thyroid gland was increased in 20–58 % of individuals, and pathological changes of the thyroid gland were found on palpation. It was stated that iodine intake is insufficient in Prague and that abnormal findings on the thyroid gland are high. Only 11.4 % of boys, 14 % of men, 6.3 % of girls and 5.4 % of women had optimal values of UIC [111].

In its final declaration in 1990, the World Summit on the Protection of Children and Their Health called for the elimination of the iodine deficit by the year 2000. The declaration was signed on behalf of the Czech Republic by the President Václav Havel. In 1993, a joint commission of WHO and UNICEF developed a strategy for the elimination of iodine deficiency in the world by the year 2000 [73].

The Interdepartmental Commission for Solving the Iodine Deficit (ICSID) was established at the National Institute of Public Health in 1995 in order to ensure cooperation between the different stakeholders involved in the control of IDD in the country. On the initiative of MUDr. B Kalvachová from the Institute of Endocrinology, in 1995 a meeting between the director of the State Health Institute Doc. MUDr. J. Kříž and Prof. MUDr. V. Zamrazil, DrSc. from the Institute of Endocrinology took place, during which the concept of solving iodine deficiency was created and the creation of the ICSID was agreed upon. Experts from medical fields (endocrinology, paediatrics, public health and hygiene), the Ministry of Health, Agriculture, Industry, Trade, non-governmental organizations and selected manufacturers of food, supplements and medicines participated in the commission’s work. The main points of solving the iodine deficiency were the promotion of higher consumption of marine products, increasing the supply of iodine with supplemented table salt and iodine-enriched foods, educating the public to understand the importance of iodine for health, and systematic monitoring and evaluation of iodine nutrition in the population [112]. MUDr. L. Ryšava, PhD from District sanitary station in Frýdek-Místek, former president of the ICSID, organized an intervention campaign, which greatly increased the number of producers of baked goods, sausages, etc., who used iodine-enriched salt for the production of their products [112]. The ICSID initiated the unification of laboratory procedures for iodine determination, their standardization and compatibility with foreign countries. ICSID member Doc. Ing. R. Bílek, CSc. from the Institute of Endocrinology became the guarantor for laboratory procedures. Prof. MUDr. O. Hníková, CSc. from the Children and Adolescent Clinic of the 3rd Faculty of Medicine of Charles University and the General University Hospital, and member of the ICSID, coordinated newborn TSH screening in the network of selected maternity hospitals [112]. Since 1999, national Iodine Days conferences have been held, and the 2002 conference concluded that the Czech Republic had fulfilled the commitment from the 1990 World Summit Declaration on Children to eliminate iodine deficiency by the year 2000 [112].

The committee has been in close contact with UNICEF and ICCIDD and worked out the program of iodine prophylaxis. Since 1995, the iodine prophylaxis program consisted of improving the iodization of table salt, when iodide could be replaced by the more thermodynamically stable iodate at a dose of 27±7 mg I/kg of salt [58,59]. The quality and precision of control of the iodine content in salt and packaging technology were improved and the expiration date of the product was introduced [34,35,59]. The use of iodized salt is not mandatory, but salt packages with the iodine logo must have an iodine content of 27±7 mg I/kg of salt [58] using iodide or iodate [36]. Legislatively, it was regulated by Decree of the Ministry of Agriculture No. 331/1997 Coll. and Decrees of the Ministry of Health of the Czech Republic No. 293/1997, No. 450/2004. According to the Decrees of the Ministry of Health of the Czech Republic No. 298/1997 and No. 446/2004, the recommended daily intake is 150 μg I. During pregnancy and breastfeeding, the need for iodine rises to 250 μg/day, and the Czech Endocrinological Society, together with the Czech Pediatric Society, recommended general iodine supplementation at a dose of 100 μg/day for all pregnant and lactating women [34, 35, 58, 59]. Infant food products are fortified with iodine [58]. The result was a substantial reduction in the prevalence of goitres and an increase in the iodine content in urine to such a value that the Czech Republic began to have an optimal supply of iodine on average [59]. Efforts to promote increased consumption of marine products, which are a significant source of iodine, should be increased [58]. However, from the middle of the first decade of this century, a rising trend of iodine levels in urine began to show (Fig. 1). One cause was an unexpected increase in iodine in cow’s milk caused by the use of iodine-fortified compound feed [113]. Changes in the iodine supply of children aged 6, 10, 13–17 years (n=1209) from Příbram and Jablonec nad Nisou showed a significant increase in average iodine levels from 140±3.69 to 221±8.66 μg/L in the period 1999–2005 [114].

Data from the Czech Republic from 2002 were published in annex C of WHO 2007 [15], which concerned 1542 participants aged 0–98 years. The mean UIC value was 129.6 μg I/L, with only 17.3 % having a UIC values lower than 100 μg I/L, which corresponded to adequate iodine intake and optimal iodine nutrition [15, 59]. In Annex G, it was stated that the Czech Republic belongs to the countries with optimal iodine nutrition, with the National Committee initialized in 1994, with a national programme and regulation, and with regular UIC and salt monitoring [15]. According to the WHO, UNICEF and lCClDD criteria, the Czech Republic has achieved the sustainable elimination of iodine deficiency as a public health problem since the year 2000 [36, 59]. Criteria for monitoring the sustainable elimination of iodine deficiency disorders are: >90 % households using adequately iodized salts, <50 % of population has UIC below 100 μg I/L, <20 % of population exists with UIC below 50 μg I/L. The multidisciplinary national programme for the elimination of IDD has been set up, a political commitment has been established to universal salt iodization and the elimination of IDD, legislation or regulations of universal salt iodization exists, and a programme of public education and social mobilization has been accepted. Regular data collection exists on salt iodine and laboratory determined urinary iodine, and a database for the recording of results is present [15]. At the end of 2004, the Czech Republic was officially included among the countries where the iodine deficit was resolved [15, 59, 73]. Between 2008 and 2023, salt iodization is voluntary in 13 countries of the WHO European Region. In six of the countries including the Czech Republic, iodine intake is documented sufficient in one or more population groups [125]. UIC studies in the WHO European Region (period 2003–2023) indicate overall adequate iodine intake in school-age children, largely due to a combination of salt iodization and iodine provided by milk and dairy products. The number of countries classified as iodine deficient based on median UIC in school-age children decreased from 23 in 2003 to two in 2023 [125]. The Czech Republic has adequate iodine nutrition in the period 2008–2023 with a median UIC in school-age children (n=400) of 248 μg I/L or in adults (n=234) of 129 μg I/L. Unfortunately, in this period, iodine nutrition 98 μg I/L is insufficient in pregnant women (n=532) [125].

## Some studies focused on iodine intake that have been conducted in the Czech Republic since 1991

A population study led by Prof. Zamrazil showed that until 1996 the average values of iodine in table salt were equal to 20 mg I/kg NaCl, but from 1996–1997 there was an increase in iodine to 34 mg/kg NaCl. The reason was the replacement of iodide with a thermodynamically more stable iodate, and there was an increase in iodine supplementation from the original 11.5–26.8 mg I/kg NaCl to 20–34 mg I/kg NaCl [35, 115]. The iodine content of table salt was determined in 1130 samples [59]. A favourable break in the supply of iodine occurred especially after 1996, when in the Czech Republic the iodization of edible salt improved both in households and in the food industry [59]. Iodized salt is used in more than 95 % of households and at least in 70 % of food manufacturing plants (bakeries, meat processing plants, dairy products, etc.) [34, 35, 59]. An improvement in the quality of production and storage of iodine-enriched salt has been achieved. Because of a number of uncontrolled sources of iodine in the diet, there is a potential risk of moving from iodine deficiency to iodine excess in some age group or categories of individuals and patients.

Periodic epidemiologic surveys were performed in 12 counties of the Czech Republic in the period of 1991–2006 [35], and the participants were randomly selected from the central register of inhabitants and later from the register of the General Health Insurance Company (VZP). There were 29612 individuals of both sexes aged 0 to 98 years, including 5263 individuals randomly selected from the general populations and 24349 individuals who attended the Institute of Endocrinology, Prague (hospital population). They provided first morning urine samples in which the UIC was measured. The median UIC progressively increased with the time, starting from values indicating mild iodine deficiency (88–95 μg I/L) prior to 1997, reaching the critical threshold of 100 μg I/L in 1998, and optimal values between 120–140 μg I/L since 2000 [59]. The average UIC in the 12 investigated areas in the period 1991 – 2006 showed that 10 % of the population is severely iodine deficient, 46 % is mildly deficient, 43 % is optimally supplied, and 1 % of the population has ioduria greater than 300 μg I/L [58]. 6 epidemiological studies were carried out five years apart in 3 regions of the Czech Republic between 1999 and 2006 (Příbram, Jablonec nad Nisou, Žďár nad Sázavou districts) [34]. The samples were selected by random selection from the register of the General Health Insurance Company. As part of the examination, thyroid parameters, nutritional status and ioduria were determined in the adult population. This was increased from a mean of 138 μg I/L (n=841) to 179 μg/L (n=789), the median increased from 113 μg I/L to 119 μg I/L. In children, the differences were even more pronounced, the mean increased from 161 μg I/L (n=974) to 228 μg/L (n=796) [34]. Side effects of increased iodine intake can lead to hypothyroidism [116] or hyperthyroidism and thyroid autoimmunity [117]. Clinically, this is probably an insignificant tendency in thyroid thyropathy, but the results indicate the need to monitor changes in population status due to changes in iodide supplementation [34].

Systematic screening for congenital hypothyroidism reveal persisting slightly elevated neonatal TSH in some parts of country [59]. The Institute of Endocrinology has not recorded an increasing number of toxic thyroid nodules or iodine-induced hyperthyroidism, although the frequency of autoimmune thyroiditis seems to have slightly increased [59]. The iodine prophylaxis has a significant positive effect on UIC, did not show an increase in abnormal thyroid function, and showed a decrease of thyroid volume in women [35].

Excessive iodine intake has been demonstrated in a number of regions and leads to an increase in the incidence of thyrotoxicosis and thyroid immunity, increases the incidence of antibodies to TPO and thyroglobulin, and may lead to a decrease in thyroid function (increase in TSH) and an increase in the volume of the thyroid gland [58]. Unfortunately, there was a significant increase in the frequency of children and adults in the category of ioduria higher than 300 μg I/L, in children from 0 % in 1999 to 23.7 % in 2004, in adults from 0 % in 1999 to 18.1 % in 2004. A certain percentage of these people may have thyroid disease, such as autoimmune thyroiditis, thyrotoxicosis or inflammation of the thyroid gland, as a result of excessive iodine intake. This is especially the case with individuals with a positive finding of antibodies against thyroid peroxidase or thyroglobulin and also in persons with polynodous goitres [59].

The most significant source of exposure to iodine in the Czech Republic in 2014–2015 was milk. Other sources included common pastries, sausages, eggs and seafood. The richest sources of iodine were infant formula, powdered soups, smoked fish and their products, meat and dairy products. The average value of iodine in milk during this period was 283 μg/kg for semi-skimmed milk and 286 μg/kg for skimmed milk [118]. In 2015, 150 so-called pool milk samples were taken from farms in 12 districts of the Czech Republic. The average iodine content of these samples was 243.7 ± 129.2 μg/L (median 243.0 μg/L). The minimum and maximum concentrations (35.0 and 688.0 μg I/L) testify to persistent significant differences in the content of iodine in milk at individual farms. Compared to the previous period, values above 1000 μg I/L were not detected [88]. Our research regarding the concentration of iodine in market milk from the Prague area was close to the recommended dose of 200 μg I/L in 2019 [119].

A survey of insured persons of the General Health Insurance Company showed an increase in thyropathies in the Czech Republic between 2012 and 2015. In women, the incidence of thyroid disease was approximately 5–6 times higher than in men. These were mainly women aged 34–45 and 65–67, and men aged 70–85. The overall prevalence rate of thyropathies increased from 6.75 % in 2012 to 7.46 % in 2015. One in five women aged 68–70 had to see an endocrinologist to treat a thyroid disorder [120].

The development of UIC of individuals visiting the Institute of Endocrinology in the period 1994–2022 is shown in Fig 1. The figure shows that iodine deficiency is not a public health problem in the Czech Republic, but it is still necessary to conduct population studies and observe a trend that should move the population from groups corresponding to iodine deficiency (0–100 μg I/L) to the group with optimal iodine intake (100–200 μg I/L), in pregnant women to the group having an intake of 150–250 μg I/L. Attention should also be paid to the group with excessive iodine intake, which has been increasing in recent years. In the group of individuals with more than adequate iodine intake (>200 μg I/L), a peak is seen with a maximum in 2003, which was probably due to the high iodine content of milk and milk products. Unfortunately, a similar increase is also visible in the years 2015–2021 (Fig. 1).

The lnstitute of Endocrinology participated in the grant of the European Commission in the frame of Horizon 2020 Stage 2-EUthyroid (Towards the elimination of iodine deficiency and preventable thyroid related diseases in Europe) proposal number 634453-Euthyroid-RlA, duration 36 months (2015–2018), together with other 22 European Union countries, lceland, Canada, Macedonia, Norway, Switzerland and lsrael. The aim of EUthyroid was to harmonize and sustainably improve iodine intake and prevent diseases associated with hypothyroidism in Europe [121]. EUthyroid generated the first harmonized data set of iodine deficiency resulting in the first valid map of the iodine status in Europe. The map demonstrates that iodine deficiency is still present particularly in adults and pregnant women in Europe, according to WHO criteria [15,122].

## Future

According to the standardized European map of median UICs, the Czech Republic belongs to the countries, where iodine deficiency is not a serious general problem in adults and school children [122]. In future, however, systematic interest should be focused on the optimization of iodide saturation, especially in pregnant or lactating women, who must have an increased supply of iodine. Children of woman suffering from insufficient iodine intake may have a decrease in intelligence quotient of 7 – 13 points and more often they develop hyperactive child syndrome [58]. The generally supported reduction in salt consumption leading to a drop in the supply of iodine is also a problem [112]. An evaluation of possible risks of excessive iodine intake must also be taken into account, as it may lead to deterioration of thyroid function including activation of thyroid autoimmunity. Systematic monitoring of iodine supplementation seems to be essential [58].

It was demonstrated that iodine deficiency is still present in Europe, using standardized data from a large number of studies. Besides the standardization of iodine monitoring studies, it will be necessary to harmonize fortification programs [122]. In Europe, iodine fortification programs differ according to type of regulation (mandatory vs. voluntary iodine fortification), amount of iodine used, and chemical form (iodine vs. iodate) [121]. Large parts of Europe can be seen as mildly to moderately iodine deficient with only 27 % of European households having access to iodized salt. Around 350 million citizens are exposed to iodine deficiency thus being at higher risk for developing neurodevelopmental anomalies [21]. Currently, there is considerable variation among countries of Europe, both in terms of iodine status and policies to address iodine deficiency [122]. 40 % of European countries (21 % of the population) have mandatory salt iodization policies, 33 % of European countries (36 % of the population) have voluntary salt iodization policies, and 27 % of countries (6 % of the population) have no data on their salt iodization policy [123].

In 2018 the Euthyroid consortium composed of experts doing long-term research in the issue of iodine supply released the Krakow Declaration on Iodine “Tasks and Responsibilities for Prevention Programs Targeting Iodine Deficiency Disorders” in response to the increasing concern about the deteriorating commitment of policymakers to address public health strategies against IDD in European populations [124]. The consortium calls on European leaders and politicians, public health officials and scientists to support action to eliminate iodine deficiency. Experts from the consortium demand that state policy and regulatory authorities harmonize the mandatory universal iodization of salt and ensure free trade in fortified foods in Europe, so that the regulation and provision of free trade is also valid for iodized feed used for fattening animals. Furthermore, the government and public administration bodies must carry out harmonized monitoring and evaluation of enrichment programs at regular intervals in order to ensure the optimal supply of iodine to the population. Also, researchers along with public health professionals, patient organizations, industry and the public should support the measures necessary to ensure the sustainability of IDD prevention programs in response to the rapidly changing environment and societal awareness of the problem [124].

Population studies examining annual UIC in approximately 400–600 randomly selected individuals of various ages from various districts of the Czech Republic will undoubtedly contribute to the knowledge of the state of the population in the area of iodine intake.
